# Electroacupuncture versus sham electroacupuncture for urinary retention in poststroke patients: study protocol for a multicenter, randomized controlled trial

**DOI:** 10.1186/s13063-016-1315-3

**Published:** 2016-04-12

**Authors:** Seungwon Shin, Jiwon Lee, Junghee Yoo, Sung Min Lim, Euiju Lee

**Affiliations:** Department of Clinical Korean Medicine, Graduate School, Kyung Hee University, 23 Kyungheedae-ro, Dongdaemun-gu, Seoul, 02447 Republic of Korea; College of Korean Medicine, Kyung Hee University, 23 Kyungheedae-ro, Dongdaemun-gu, Seoul, 02447 Republic of Korea; Department of Motor & Cognition Rehabilitation, Korean National Rehabilitation Research Institute, 111 Gaorigil, Gangbuk-gu, Seoul, 01022 Republic of Korea

**Keywords:** Stroke, Cerebrovascular accident, Urinary retention, Electroacupuncture, Randomized Controlled Trial

## Abstract

**Background:**

This study protocol evaluates the effectiveness of adjuvant electroacupuncture (EA) for urinary retention in poststroke patients undergoing conventional treatments, in comparison with that of a sham control.

**Methods/design:**

A multicenter, blinded, randomized controlled trial will be conducted in three hospitals in the Republic of Korea. We are recruiting 54 stroke survivors (aged >19 years), who were diagnosed with urinary retention based on the results of two consecutive post-void residual (PVR) tests, and dividing them randomly into two arms: the EA and Park-sham control groups.

They will receive ten sessions of EA or sham treatment for 2 weeks. The participants will be blinded with non-penetrating needles and fake sounds of EA stimulators. The daily PVR ratio will be primarily measured at baseline and at the end of the study to statistically test the effectiveness of EA for poststroke urinary retention. Then, the Korean version of the Qualiveen Questionnaire, the Korean version of the International Prostate Symptom Score, and the blinding index will be assessed. After each EA session or sham EA, adverse events will be reported to evaluate the safety of EA.

Results will be analyzed by using the independent *t*-test or Mann-Whitney *U* test, based on both intention-to-treat and per-protocol principles.

**Discussion:**

The findings will provide clinical evidence for the effectiveness of EA treatment to improve urinary retention in stroke survivors.

**Trial Registration:**

This study protocol was registered in ClinicalTrials.gov (NCT02472288) on 10 June 2015.

## Background

Urinary retention (UR) or incomplete bladder emptying (IBE), defined as a post-void residual (PVR) urine volume of >100 mL on two consecutive occasions [1, 2], occurs in approximately 29 % [1] to 56 % [3] of poststroke patients. Bladder problems that occur after stroke episodes deteriorate the stroke survivors’ quality of life (QoL) and affect their disability and mortality [4–6]. UR in stroke survivors is usually managed with catheterization, but this procedure interferes with their active rehabilitation and can induce urinary tract infection (UTI) [2]. In the long term, UR without adequate management can lead to renal dysfunction [7]. Thus, caregivers should provide poststroke patients who have UR with proper bladder management to improve the patients’ functional outcomes [8].

In the United Kingdom, sacral neurostimulation has been known to be effective for UR induced by urethral sphincter overactivity (Fowler’s syndrome). However, significant complications have occurred, such as inserted lead migration or high surgical revision rates [9]. Bladder problems have also been a concern of traditional medicine practitioners. Diabetic bladder dysfunction was improved with acupuncture treatment in a pilot study [10]. Another randomized controlled trial showed that, besides sham electroacupuncture (EA), EA is an effective treatment of bladder muscle overactivity in poststroke patients [11]. A small-scale of clinical study with a single study group suggested the potential benefit of EA therapy for poststroke patients with IBE.

However, randomized controlled trials designed with adequate statistical power and sham comparators should be conducted to determine whether EA is effective for poststroke UR. We will conduct a multicenter, randomized controlled trial to evaluate the effectiveness of adjuvant EA therapy for poststroke patients with UR who are undergoing conventional treatments, in comparison with that of sham EA.

## Methods/design

### Study design

**Fig. 1:**
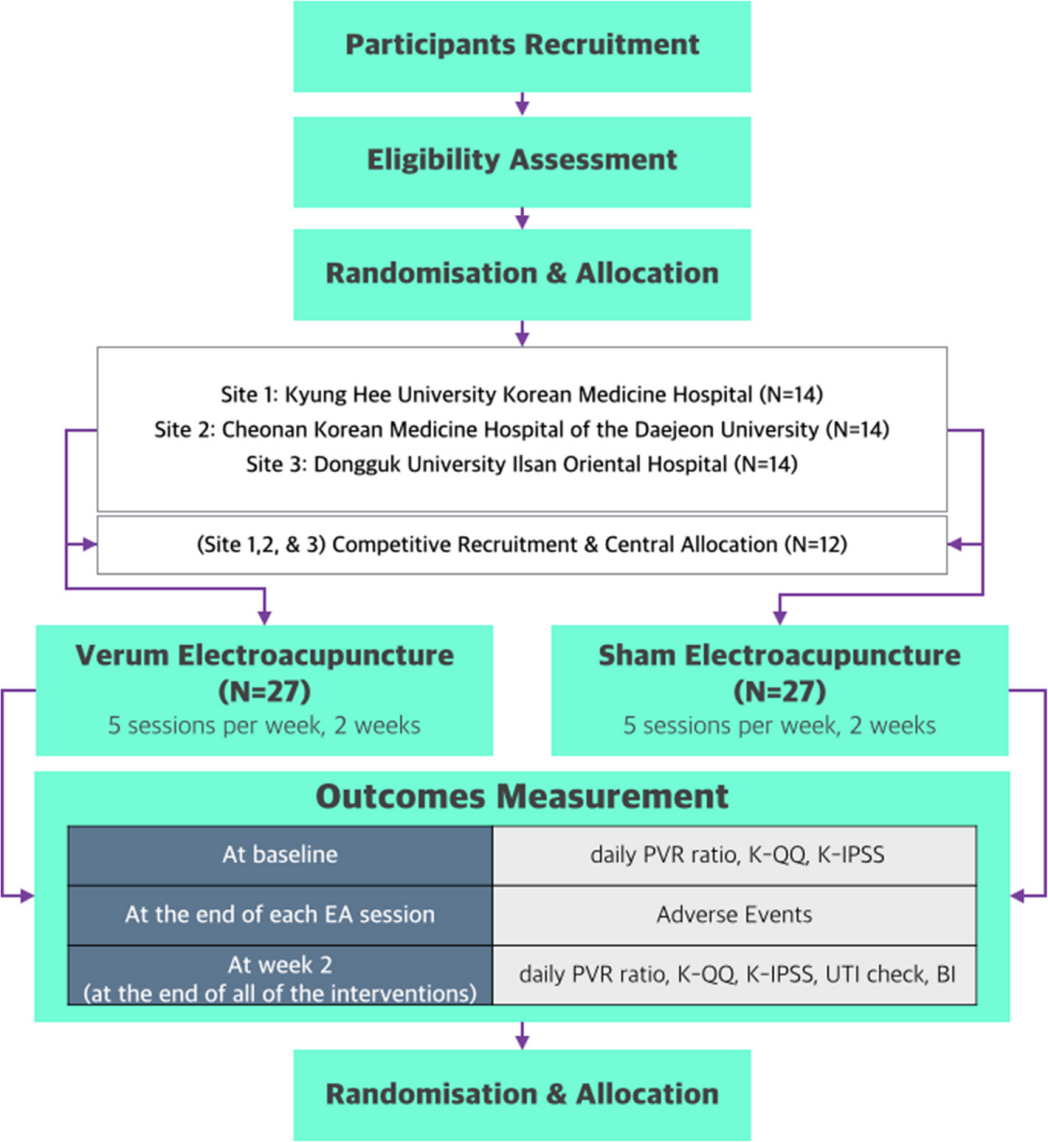
Flowchart of the study process. Fifty-four participants will be randomized and allocated to either the intervention or control group. After conducting ten sessions of verum or sham electroacupuncture for 2 weeks and examining for urinary tract infection (UTI), the daily post-void residual (PVR) ratio, Korean version of Qualiveen Questionnaire score (K-QQ), Korean version of the International Prostate Symptom Score (K-IPSS), and blinding index (BI) will be measured. Adverse events will be investigated right after each session

This is a multicenter, blinded, randomized controlled trial to compare an EA group with a sham EA group, with an allocation ratio of 1:1. We designed the protocol, conforming to the Consolidated Standards of Reporting Trials (CONSORT) [12] and Standards for Reporting Interventions in Clinical Trials of Acupuncture (STRICTA) [13] guidelines. The study process is illustrated in Fig. 1. The institutional review board of Kyung Hee University Korean Medicine Hospital approved the study (KOMCIRB-150213-HR-005). The protocol has been registered in ClinicalTrial.gov (NCT02472288).

### Participants

We will recruit 54 male or female patients aged ≥19 years from three hospitals in Korea: Kyung Hee Korean Medicine Hospital in Seoul, Cheonan Korean Medicine Hospital of Daejeon University in Cheonan, and Dongguk University Ilsan Oriental Hospital in Ilsan. In order to identify survivors from stroke episodes, like intracerebral hemorrhage or cerebral infarction, experienced practitioners will check the PVR urine volume twice a day. The patients will be pre-screened only if both residual volumes are >100 mL. After we obtain informed consent from the patients or their representatives, we will screen them according to the eligibility criteria for enrollment in the study.

### Inclusion criteria

We will include both male and female patients aged >19 years whose stroke episodes occurred within the last 2 years. We will not restrict inclusion of participants according to type of stroke or damaged region in the brain. Stroke history will be confirmed based on computed tomography or magnetic resonance imaging examination results. Two consecutive PVR tests will be performed, and the patient with the results of both residuals > 100 mL will be included in the trial [1, 2].

### Exclusion criteria

We will exclude stroke patients with any one of the following clinical conditions from the medical screening process: (1) bleeding disorders, (2) procedures or surgeries for peripheral vascular diseases, (3) psychiatric disorders, (4) severe diseases in the lower urinary tract, (5) acute or chronic infectious diseases in the lower urinary tract, or (6) pregnancy (laboratory results from urinalysis). In addition, patients (7) whose medications for UR or relevant symptoms (urinary incontinence drugs or diuretics) have been changed within 3 days or (8) for whom onset of treatment is within 1 week prior to the study, along with those with a Glasgow Coma Scale score of <8 points, will be excluded. Finally, we will also exclude those who have fears about acupuncture therapy.

### Dropout criteria

Patients will be removed from the study if they are unwilling to continue their participation in the study. Patients who fail or anticipate to fail in continuing at least seven sessions will be dropped from the study, too.

### Randomization

Random sequences were generated separately in each site with the R package (R Foundation for Statistical Computing, Vienna, Austria, https://www.r-project.org) to allocate participants into either the EA or sham group. The allocation ratio was 1:1. From each center, at least 14 participants will be recruited, and the last 12 persons will be competitively enrolled among all the three sites. Only the delegated sub-investigators can access the security electronic file of the randomization table and assign the participants. Practitioners from each site will call or send a message to the sub-investigators in order to allocate the participants in their own hospitals. After assignments are completed, a sub-investigator from the primary site, Kyung Hee University Korean Medicine Hospital, will allocate the 12 participants from the three sites to the last participant. The investigators, who will generate the random number and assignments, will be blinded to the arm where each participant will be allocated.

### Blinding

The patients and outcome assessors will remain blinded during the study. Patients assigned to the sham group will undergo a similar treatment as those assigned to the EA group. Because we will use the Park-sham with non-penetrating acupuncture needles as control, electrostimulation will be turned on without conductance. In addition, we will use the same guide tubes used in the Park-sham acupuncture needles in the intervention group, too. Aside from the practitioners who will administer the EA or sham treatment, there will be an independent outcome assessor who will not be notified about the patients’ allocated arms.

### Interventions

The EA and sham EA regimens are based on traditional Korean medicine. We referred to the textbook [14] and consulted acupuncture and stroke specialists.

**Fig. 2:**
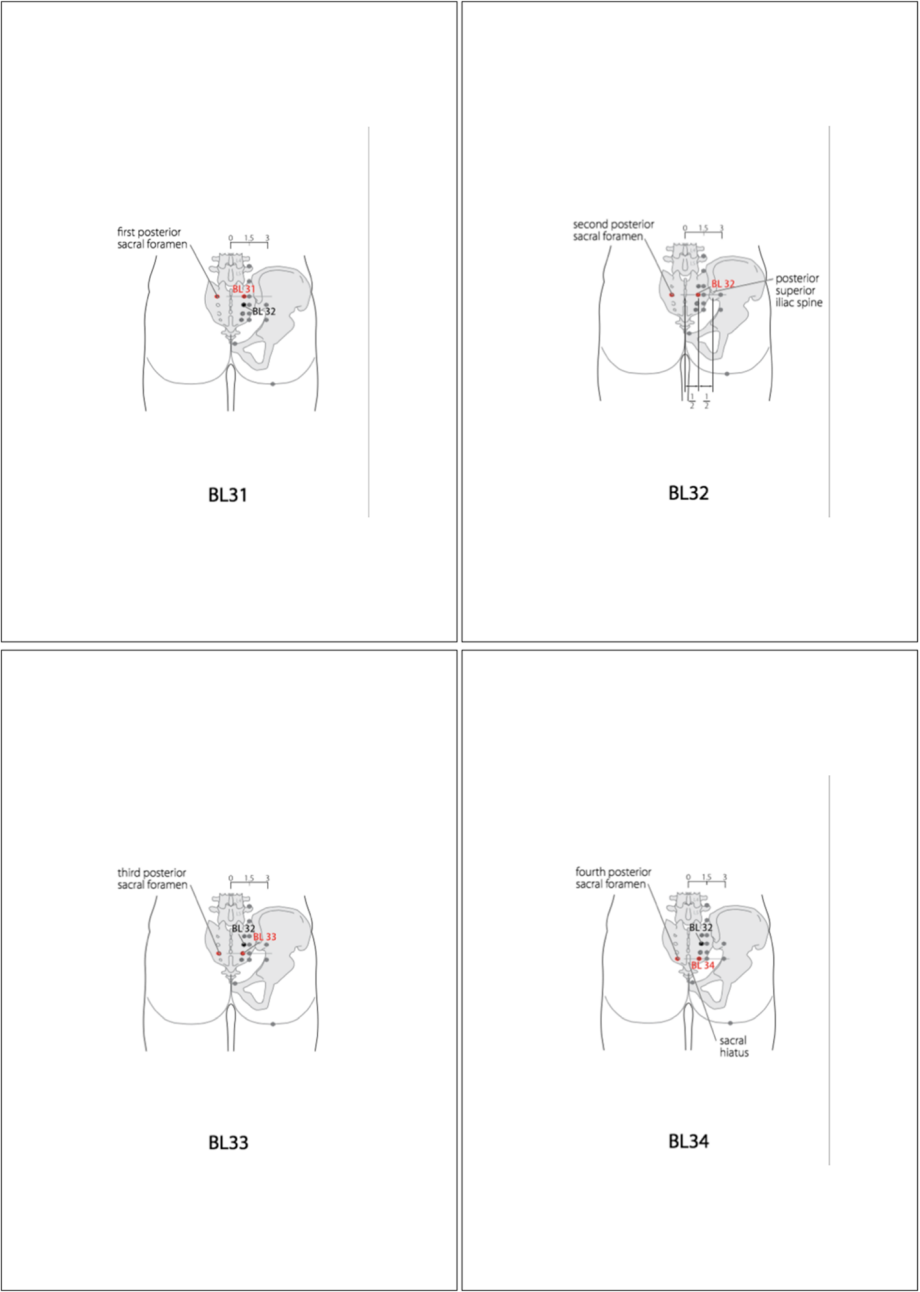
The locations of acupoints BL31, BL32, BL33, and BL34. In the sacral region, BL31, BL32, BL33, and BL34 are located in the first, second, third, and fourth posterior sacral foramens, respectively. (World Health Organization Standard Acupuncture Point Locations in the Western Pacific Region)

The participants allocated to the EA group will receive five sessions per week of EA therapy for 2 weeks (ten sessions in total). After inserting needles (stainless steel, 0.25 mm in diameter and 4.0 mm in length; Dong Bang Acupuncture Inc., Republic of Korea) using the Park-sham guide tube on bilateral acupoints BL31, BL32, BL33, and BL34 (eight points in total) at 5–10 mm in depth, the *de qi* sensation is elicited. The locations of the acupoints according to the World Health Organization Standard Acupuncture Point Locations in the Western Pacific Region are illustrated in Fig. 2 [15]. *De qi* means an acupuncture needle sensation of soreness, tingling, fullness, ache, coolness, warmth, and heaviness, and a radiating sensation at and around the acupoints to the point of a threshold that elicits nerve impulse transmission to the cerebral cortex [16]. Then, electrostimulation will be administered for 20 minutes at a moderate frequency of 30 Hz (STN-111, Stratek, Republic of Korea).

We will use the Park-sham device in the control group. The non-penetrating needles of the sham acupuncture will be implemented on the same eight bilateral acupoints. The total number of sessions of sham EA is ten (five times per week for 2 weeks). The electrostimulation will also be administered for 20 minutes at moderate frequency (30 Hz; STN-111, Stratek, Republic of Korea), even though the stimulation will not be delivered through the skin because of the non-penetrating needle of sham device.

The use of conventional therapies will be allowed during the study for both groups. The conventional therapies consist of general supportive care, preventive or therapeutic management for medical/neurological complications, or drug treatments with antiplatelet agents, anticoagulants, or neuroprotectants. Moreover, stroke management with traditional herbal medications, acupuncture without electrostimulation, or rehabilitation for stroke complications will be permitted during the trial. However, any drugs, herbal medications, or rehabilitation that could affect UR will be prohibited. The study will conform to clinical practice guidelines for stroke management in Korea [17].

Practitioners who have over 1 year of clinical experience will perform the EA or sham EA treatments.

### Primary outcome

To evaluate the effectiveness of EA treatment over the sham device for poststroke UR, we will primarily assess the change in daily PVR urine ratios between the baseline and end point of the last session. The daily PVR ratio is computed by dividing the daily PVR urine volume by the daily PVR urine volume plus spontaneous voiding volume. The daily PVR urine volume for 24 hours will be examined with ultrasonography scanning. If the PVR urine volume is more than 100 mL, the residual will be removed through intermittent cauterization before the scanning. When spontaneous urination is impossible, a 24-hour weight change in diaper will be measured for the self-voiding volume.

If the urinary tract is infected at the end, the outcome assessor will measure the PVR urine volume on the third day of the last session after anti-UTI treatment for 48 hours. Otherwise, PVR urine volume will be measured on the next day of the last session.

### Secondary outcome

Because UTI might affect the urine residuals [18, 19], the patients in both groups will undergo urinalysis to determine whether UTI exists before PVR urine volume evaluation at the end point. UTI is diagnosed when nitrite is detected by urinalysis and two of the following clinical symptoms exist: pyuria, lower abdominal pain, or hematuria [20]. The urinalysis retest is performed after 48 hours of antibiotics treatment.

We will also assess disease-specific QoL by using the Korean version of the Qualiveen Questionnaire (K-QQ). This is an assessment tool consisting of 30 items for patients with neurogenic bladder [21–24]. Each item, rated from 0 to 4 points, evaluates patients’ bladder problems, while the additive nine questions, rated from −2 to +2 points, are about patients’ general QoL status. The higher the absolute value of the total K-QQ score, the lower the QoL becomes. We will measure the K-QQ scores at baseline and at the end of the last session.

The Korean version of the International Prostate Symptom Score will be assessed at baseline and at the end point. This scale is generally used to evaluate general symptoms related to the lower urinary tract [25, 26]. The total score (0–35) is calculated by seven items of 0–5 points each, and the severity will be assessed as mild (0–7 points), moderate (8–19 points), or severe (20–35 points).

The frequencies of urination and urinary incontinence will also be reported. The patients will record them by urinary diary for 1 day before the first intervention and for another day after the last intervention. The mean change from posttreatment to pretreatment in the EA group will be compared with that in the sham group.

Finally, we will measure the blinding index (BI) for all of the participants at the end of the verum or the sham EA. The success or failure of the participants’ blinding will be assessed by using the validated scale [27]. The outcome assessor will ask the participants where they belong by selecting one of the following responses: “I think I belonged to the EA group,” “I think I belonged to the sham group,” or “I don’t know which group I belonged to.”

The patient-rated outcomes will not be measured when the participants are unconscious. The two caregivers with more than 1 year of practical experience, after being educated about the scales, will independently fill in the scales for the participants with low cognitive function and the discrepancy will be settled through discussion.

### Other outcomes

Information on diabetes mellitus (DM) and benign prostatic hyperplasia (BPH) will be obtained from the participants’ medical history.

### Safety assessment

Adverse events will be reported to evaluate the safety of EA for poststroke patients with UR. The questionnaires will be administered to the participants after every verum or sham EA session, and the results will be recorded and analyzed. The outcome assessors will judge the severity (mild, moderate, or severe), seriousness, and causality (definitely related, probably related, possibly related, possibly not related, definitely not related to the intervention, or not assessable).

### Sample size

Based on a previous study [2], the mean percentage change in PVR ratios in the EA and control groups were computed. We assumed an after-treatment PVR ratio of 0.65 in the control group, as it was not reported in the study. The null hypothesis was that the percentage changes in PVR ratios have no significant difference between the intervention and control groups. With a significance level of 0.05 and a statistical power of 0.8, the calculated sample size in total was 42 [28]. Considering a 20 % drop rate at a 1:1 allocation ratio, we will recruit 27 participants for each group.

### Data analysis

We will analyze results primarily based on the principle of intention - to - treat (ITT) and subordinately based on the per - protocol (PP) principle. The ITT analysis will include any participants who are randomly allocated and have at least one EA or sham session. Missing values will be imputed by the last observation carried forward. The PP analysis will cover only the patients who have at least seven sessions of EA or sham treatments and have no medical diagnosis of UTI at the end point.

Continuous variables will be tested by the independent *t*-test if normality is obtained. Otherwise, the Mann-Whitney *U* test will be implemented. The categorical variables will be analyzed by the Pearson *χ*^2^ test or Fisher's exact test.

## Discussion

This randomized controlled trial aims to determine whether EA treatment is effective for UR in poststroke survivors. The effectiveness is represented by the relative proportion of urine residuals and voiding volumes. Because this study is designed to provide clinical evidence for the relevant patients and practitioners, we have chosen not to eliminate any other general treatments for poststroke survivors.

The eight acupoints were identified based on the guideline published by the World Health Organization. In the sacral region, BL31, BL32, BL33, and BL34 are located in the first, second, third, and fourth posterior sacral foramens, respectively [15]. In the literature on traditional Korean medicine, these points have been reported to possibly have the property to recover bladder dysfunction, and they have shown clinical effectiveness for poststroke detrusor overactivity in a previous study [11]. The acupoints are considered to have a possible mechanism that the acupuncture stimulation directly increases the excitability of the pelvic nerve, which consequently innervates the detrusor muscle [2].

Park-sham acupuncture is a placebo acupuncture device invented to stabilize the needle in position with an additional guide tube preventing skin penetration [29]. This sham device was validated to be inactive and indistinguishable [30]. We have decided to use the same guide tubes as Park-sham acupuncture to prevent participants from discovering the group to which they belong. The apparatus for electrostimulation will be activated in the sham control group to make the participants hear the same machine sound so as to let them believe they are under real acupuncture treatment. Because Park-sham acupuncture does not intrude the skin, electrostimulation will not be conducted through the body. Success or failure of these blinding strategies will be appraised at the end of the study by the BI [26].

Bladder dysfunction, including UR, poses an obstacle for rehabilitation and QoL improvement in poststroke patients [2, 4–6]. Catheterization, the major therapeutic approach, confers the risk of UTI [2]. A preliminary clinical study showed the potential benefits and safety of EA treatment for stroke patients with UR [2]. However, the study was performed only with a single group and lacked the methodological requirements and statistical power to establish the evidence for treatment. Overall, clinical trials of EA for any urinary dysfunction, with a placebo control, are rare, except for the study of poststroke detrusor overactivity [11]. We targeted UR in poststroke patients and designed a randomized, sham-controlled trial to be conducted in multiple sites to appropriately generalize the results of the trial, following the CONSORT [12] and STRICTA guidelines [13].

At the end of the project, we expect that the findings will provide clinical evidence of the effectiveness of EA treatment for improving UR in stroke survivors.

## Abbreviations

BI: blinding index
BPH: benign prostatic hyperplasia
DM: diabetes mellitus
EA: electroacupuncture
IBE: incomplete bladder emptying
ITT: intention to treat
K-QQ: Korean version of Qualiveen Questionnaire
PP: per protocol
PVR: post-void residual
QoL: quality of life
UR: urinary retention
UTI: urinary tract infection
